# Planar Perovskite Solar Cells Using Perovskite CsPbI_3_ Quantum Dots as Efficient Hole Transporting Layers

**DOI:** 10.3390/ma15248902

**Published:** 2022-12-13

**Authors:** Tsair-Chun Liang, Hsin-Yu Su, Sih-An Chen, Yen-Ju Chen, Chung-Yu Chiang, Chih-Hsun Chiang, Tzung-Ta Kao, Lung-Chien Chen, Chun-Cheng Lin

**Affiliations:** 1 Institute of Photonics Engineering, National Kaohsiung University of Science and Technology, Kaohsiung 824005, Taiwan; 2 Department of Electro-Optical Engineering, National Taipei University of Technology, Taipei City 106344, Taiwan; 3 Department of Mathematic and Physical Sciences, R.O.C. Air Force Academy, Kaohsiung 820008, Taiwan; 4 Department of Electronic Engineering, Ming Chi University of Technology, New Taipei City 243303, Taiwan

**Keywords:** perovskite, quantum-dot (QD), nanoparticle (NP), perovskite solar cell (PSC)

## Abstract

Perovskite CsPbI_3_ quantum dots (QDs) were synthesized as a hole-transporting layer (HTL) of a planar perovskite solar cell (PSC). By using the Octam solution during the ligand engineering, CsPbI_3_ QDs exhibits a denser grain and a larger grain size due to the short-chain ligands of Octam. In addition, CsPbI_3_ QDs with the Octam solution showed a smooth and uniform surface on MAPbI_3_ film, indicating the QDs improved the microstructure of the MAPbI_3_ perovskite film. As a result, the PSC with CsPbI_3_ QDs as an HTL has the optimal open-circuit voltage as 1.09 V, the short-circuit current as 20.5 mA/cm^2^, and the fill factor (FF) as 75.7%, and the power conversion efficiency (PCE) as 17.0%. Hence, it is inferred that introducing QDs as a HTL via the ligand engineering can effectively improve the device performance of the PSC.

## 1. Introduction

Economic growth and industrialization have led to increased use of fossil fuels. Increased production and consumption of fossil fuels has had several adverse environmental impacts on the countries, including global warming, air pollution and increased health risks [1]. Therefore, the promotion of renewable energy is imperative and the International Energy Agency (IEA) released a roadmap for realizing net-zero carbon dioxide (CO_2_) emissions in the energy sector by 2050 [2]. Among renewable energies, solar generation rose 23% and wind by 14% in 2021. Aggregately, this takes them to more than 10% of global electricity generation. All clean electricity sources generated 38% of the world’s electricity, more than coal (36%) [3].

In order to increase the total amount of power generation, only increasing the power generation device or improving the energy conversion of the device is insufficient. Perovskite solar cells (PSCs) are photovoltaic (PV) devices capable of converting abundant sunlight radiation into electricity, and they are expected to accelerate the development of renewable energy because of their high conversion efficiency, low production cost, and ease of fabrication [4,5]. In 2009, Kojima et al. pointed out the use of organic–inorganic halide perovskites such as CH_3_NH_3_PbBr_3_ (MAPbBr_3_) and CH_3_NH_3_PbI_3_ (MAPbI_3_) to enhance the photovoltaic effect in photo electrochemical cells (PECs), with power conversion efficiencies (PCE) of 3.1% and 3.8%, respectively [6], hence, attracting the interest of many researchers around the world, allowing the PCE of PSC to rapidly increase to 25.5% [7].

Optimizing electron and hole-transport materials (ETM and HTM) is a key factor in improving the performance of solar cell devices [8]. In the past, many studies put much effort into optimizing the interface using different materials [9,10,11,12]. Recently, perovskite quantum dots (QDs) have become an alternative HTM, such as the insertion of FAPbX_3_ perovskite quantum dot layers to enhance the PCE of PSCs [13]. Subsequently, the perovskite CsPbI_3_ QDs, which has the same structure as the emissive layer, has also been investigated as HTL due to its matching energy, high moisture stability [14], and the better stability of all-inorganic materials than organic ones in perovskites [15]. In addition, synthesis methods and ligand exchange engineering for inorganic perovskite CsPbX_3_ QDs have been widely studied to improve the photoelectric properties of QDs [16]. However, the ligand exchange of perovskite QD-based HTM for improving interfacial carrier transporting of perovskite solar cells has not yet been discussed.

Accordingly, this study proposes a strategy to optimize interfacial engineering using ligand engineering by replacing the long-chain ligand Oleylamine (OAM) with the short-chain ligand Octylamine (Octam) and introducing the improved QDs into PSCs, thereby improving efficient carrier transport [17,18]. The result showed that QDs with interfacial engineering had higher PCEs compared to PSCs without the interfacial engineering of QDs. This study illustrates an approach to achieve a higher efficiency of PSC.

## 2. Experimental Section

### 2.1. Materials

Cesium carbonate (Cs_2_CO_3_), methyl acetate (MeoAc), and octane (OCT) were acquired from Alfa Aesar. Octadecene (ODE), and oleic acid (OA), Octam, PbI_2_, tetraoctylammonium bromide (TOAB), hexane, and OAM were purchased from Sigma-Aldrich. All other materials and solvents were received from Echo Chemical Co. Ltd. (Miaoli, Taiwan). Besides, CH_3_NH_3_I and ZnO were synthesized using the same method previously published in the literature [19,20].

### 2.2. Synthesis of CsPbI_3_ QD Solution

Measures of 320 mg of Cs_2_CO_3_, 9 mL of ODE, and 0.75 mL of OA were loaded into a 50 mL glass bottle and then the solution was heated to dissolve completely using a hotplate stirrer and was vacuumed during the process. Measures of 0.17 g of PbI_2_ and 10 mL of ODE were loaded into a glass bottle, and then the solution was heated to dissolve completely with a hotplate stirrer and vacuumed during the process. Then, 1 mL of OA and 1 mL of OAM (or Octam) were injected into the solution and heated to 150 °C for 5 min to prepare CsPbI_3_ QDs with OAM ligands (or the short-chain Octam ligands). Afterwards, 0.8 mL of cesium oleate precursor was added into the solution by the rapid hot-injection method and cooled in an ice-water bath to synthesize CsPbI_3_ quantum dots. As-prepared solution was added to MeoAc with a volume ratio of 1:1 and centrifuged at 12,000× *g* rpm for 10 min. The powder precipitated at the bottom was added to 1 mL of hexane and 3 mL of MeoAc and centrifuged at 12,000× *g* rpm for 10 min. Once again, the powder precipitated at the bottom was added to 1 mL of OCT and centrifuged at 12,000× *g* rpm for 10 min. Finally, the supernatant was stored at 4 °C until further use.

### 2.3. Device Fabrication

The sol–gel ZnO precursor solution was spin-coated at 5500 rpm for 30 s and annealed at 140 °C for 30 min. Then, the MAPbI_3_ perovskite layer was deposited by spin-coating PbI_2_ dissolved in dimethylformamide (DMF) (1 mole/L) onto the ZnO layer at 3000 rpm. As the solution dried, CH_3_NH_3_I solution was spin-coated onto PbI_2_ at 3000 rpm. More detailed information about the two-step spin coating method has been reported in [21]. Then, CsPbI_3_ was spin-coated onto the MAPbI_3_ perovskite layer at 2000 rpm for 20 s prior to annealing at 100 °C for 20 s. Afterwards, the sample was successively deposited with molybdenum trioxide (MoO_3_) and Ag cathode by thermal evaporation technique in a high vacuum chamber. The film thickness and deposition rate for the above materials were 5 nm at 0.3 nm/s and 100 nm at 1.5 nm/s, respectively. Eventually, the PSCs were fabricated and encapsulated with a UV-light curing adhesive in a nitrogen-filled glovebox.

### 2.4. Measuring Instruments

The photoluminescence (PL) spectra were measured by a fluorescence spectrophotometer (F-7000, Hitachi, Minato City, Tokyo, Japan). The time-resolved photoluminescence (TRPL) decays were obtained using a HORIBA FluoroMax Plus spectrofluorometer. J-V characteristics.

Solar simulator and EQE spectra were recorded using SS-X Solar system (Enlitech, Kaohsiung, Taiwan) and QE-R quantum efficiency system (Enlitech), respectively. The XRD patterns were analyzed using an X-ray diffractometer (D8 advance, Bruker, Ettlingen, Germany). TEM images with a resolution of ≤0.23 nm were obtained using a JJ JEM-2100 Plus microscope at an accelerating voltage of 100 kV. The atomic force microscope (AFM) was measured by the tapping mode (INNOVA AFM, Bruker) and has nanometer resolution in the *x*-*y* plane and angstrom resolution in the *z*-axis.

## 3. Results and Discussion

**Figure 1:**
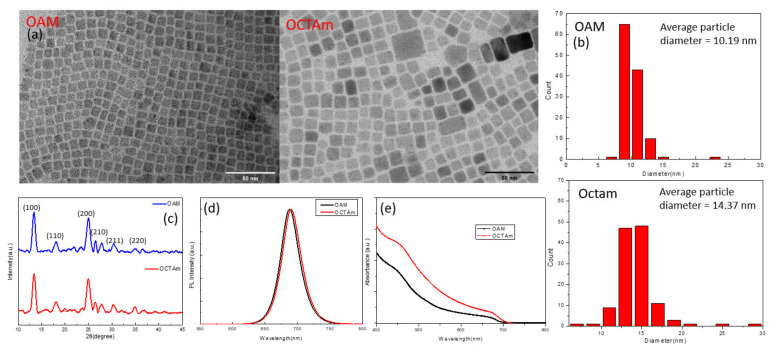
(**a**) TEM images and (**b**) size distribution for QD films; (**c**) XRD pattern of CsPbI_3_ QDs; (**d**) PL spectrum and; (**e**) UV–vis absorbance spectra for QD films.

In this study, the above synthetic method was used to prepare samples of two CsPbI_3_ QD solutions. One is CsPbI_3_ QDs with OAM ligands, and the other is CsPbI_3_ QDs with Octam ligands through ligand engineering. Both CsPbI_3_ QD solutions were purified three times to obtain high quality QDs with a photoluminescence quantum yield (PLQY) of 40.2% and 38.9% for OAM-ligand and Octam-ligand QDs, respectively. To observe the characteristics of the QDs more closely, transmission electron microscopy (TEM) was used to measure the morphology of both QD solutions. Figure 1a shows the crystalline form of QDs with a cubic shape and no aggregation phenomenon was found. Figure 1b presents the size distribution of QDs with OAM and Octam ligands, with standard deviations of 2.3 nm and 3.0 nm, respectively, implying that the QD size distribution is uniform. The average diameters of CsPbI_3_ QDs with OAM and Octam ligands were 10.19 nm and 14.37 nm, respectively, and the difference in size was attributed to ligand engineering by replacing the long-chain ligands (OAM) with the short-chain ligands (Octam), resulting in the larger size [22].

In addition, the QD structure was further confirmed as a cubic morphology by X-ray diffraction (XRD), and the peak position of the XRD pattern in Figure 1c is consistent with previous study [23]. Figure 1d illustrated the photoluminescence (PL) emission spectrum of the QD solutions and showed the emission peaks at 685 nm (OCTAM ligands) and 690 nm (OAM ligands), indicating that the CsPbI_3_ QDs were obtained using both ligands. The CsPbI_3_ QDs with OCTAm ligands are slightly red-shifted compared to the CsPbI_3_ QDs with OAM ligands in the PL spectrum, which is caused by the size-up of the QDs and corresponds to the TEM images [24]. However, the ultraviolet–visible (UV–vis) absorbance spectra in Figure 1e exhibit that both QD solutions are absorbed in the visible spectrum, which can enhance the light absorption of PSC [13]. The band gaps for the OAM- and OCTAM-based CsPbI_3_ QDs can be calculated as 1.84 eV and 1.85 eV through the UV-vis absorbance spectra (see Supplementary Materials Figure S1), respectively, which is also in agreement with the previous study [25,26].

**Figure 2:**
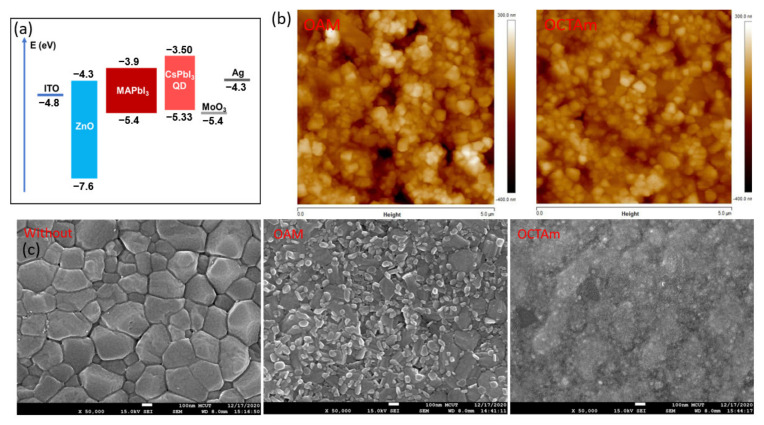
(**a**) The energy level diagram of PSC; (**b**) SEM and (**c**) AFM images of MAPbI_3_ films.

According to the results of the above analyses, the high-quality QD solutions were synthesized and then introduced the QDs between HTM and perovskite film for PSCs by interfacial engineering. In the study, the schematic structure of the PSC is shown in Figure 2a, which consists of a multilayer structure ITO/ZnO/MAPbI_3_/QD/MoO_3_/Ag. For further investigation of the QDs on MAPbI_3_ perovskite film, a scanning electron microscope (SEM) was used to measure MAPbI_3_ before and after being coated with the QDs and found that MAPbI_3_ perovskite film without QDs showed a clear crystal structure, as shown in Figure 2b. However, the QDs using OAM ligands appear granular on the MAPbI_3_ perovskite film in Figure 2b. In contrast, the QDs using Octam ligands showed a smooth and uniform surface of MAPbI_3_ film. A similar observation was obtained in the atomic force microscope (AFM) measurement in Figure 2c, which is identical to the SEM images in Figure 2b. The QDs using Octam ligands showed a uniform distribution on MAPbI_3_ perovskite film compared to the QDs using OAM ligands, and their root-mean-square roughness (*S*_q_) was 57 nm and 83 nm, respectively.

It is well known the matching energy levels can optimize the performance of the PSCs [27]. Therefore, in this study, the energy-level matched QDs between MoO_3_ (hole injection layer) [28] and perovskite MAPbI3 films were introduced through interfacial engineering to increase the hole transfer without significant energy loss in order to improve the efficiency in the hole-transport layer, as shown in Figure 2a [29]. To verify the effect of QDs on PSC and the importance of matching energy levels, this study experimentally fabricated three types of PSCs, one without QD coating, another with QDs using OAM ligands, and the other with QDs using Octam ligands. Interestingly, the PSC coated with the QDs using OAM ligands failed in measurement, implying that this PSC cannot conduct current. This phenomenon can be attributed to two possibilities. One is that the QDs using long-chain insulating ligands hinder carrier transport in device applications [30]. The other possibility is that the QD coating cannot form a film on the MAPbI_3_ layer, leading to an increase in surface defects, resulting in defects of trap free charges and reducing the current density, leading to a lower luminance [31]. This result also matches with Figure 2b. Consequently, the traditional long-chain OAM ligand on the QDs cannot form a full coverage film on the MAPbI_3_ layers, but the short-chain Octam ligands can be used as the suitable HTM. In this way, the disadvantage of conductivity can be solved and the formation of the surface film can be more uniform and smoother in Figure 2c, thus improving the efficiency of hole transport through ligand engineering.

**Figure 3:**
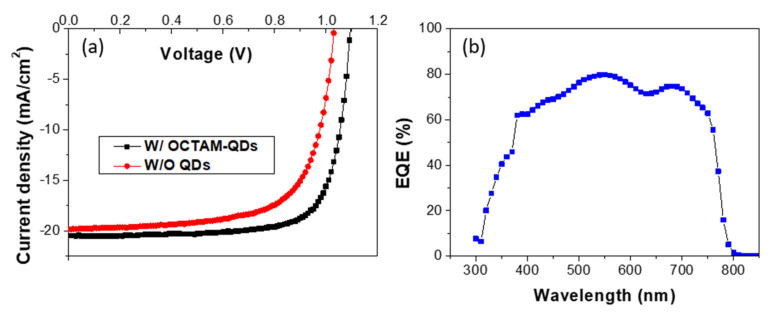
(**a**) J-V characteristics for PSCs and (**b**) EQE spectrum of the PSC with QDs using Octam ligands.

Figure 3a presents the current-voltage (J-V) characteristics for the PSC without QD coating and the one coated with QDs using Octam ligands. It is shown that the PSC coated with QDs using Octam ligands exhibited significant improvement in photovoltaic performance. The open-circuit voltage increased from 1.03 to 1.09 volts, the short-circuit current increased from 19.8 to 20.5 mA/cm^2^, and the fill factor (FF) increased from 68.7% to 75.7%, corresponding to an increase in power conversion efficiency (PCE) from 14.1% to 17.0%. It can be seen that introducing QDs enhances the carrier mobility through the interfacial engineering to make the energy levels matching, so that PSC with QDs using Octam ligands can obtain higher open-circuit voltage, short-circuit current, and further improve the PCE. Figure 3b showed the EQE spectrum of the PSC with QDs using Octam ligands, where the maximum EQE of that reached 79.7%.

**Figure 4:**
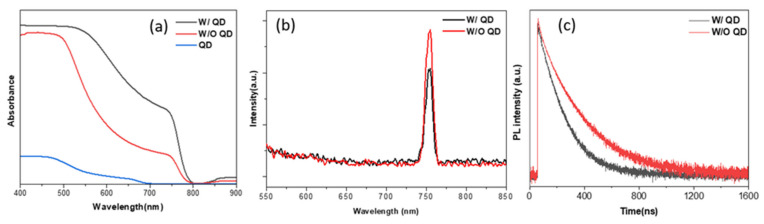
(**a**) The absorbance spectra; (**b**) PL spectrum; and (**c**) TRPL decay curves for MAPbI_3_ films.

For further examination of the photovoltaic performance of PSC improved by introducing QDs, the absorbance of MAPbI_3_ films with and without QDs was measured, and it was found that the absorbance of the film with QDs was significantly enhanced over that of the film without QDs in the range from 500 nm to 750 nm. Similarly, QDs also showed a slight signal in the absorbance between 500 nm to 700 nm in Figure 4a. Furthermore, the PL spectra of the MAPbI_3_ films with and without QDs were measured in Figure 4b to demonstrate that introducing QDs improved the charge transfer, and there was no obvious shift in the peak position, but a difference in PL intensity. The PL intensity of MAPbI_3_ film with QDs was 30% lower than that without QDs, which could be attributed to the charge carrier extraction enhanced by the introduction of QDs [14]. We carried out the time-resolved photoluminescence (TRPL) measurement to investigate the effect of QDs on the hole extraction kinetics at the interface between MAPbI_3_ and HTM layers. Figure 4c showed the decay lifetime of the MAPBI_3_ film without QDs is 110 ns, but that of the MAPBI_3_ film with QDs is greatly shortened to 88 ns, indicating a rapid charge transfer from perovskite material to HTM [32]. As a result, the PSCs with QDs possesses better performance in PCE than those without QDs.

## 4. Conclusions

This study has examined the effects of perovskite CsPbI_3_ QDs via the ligand engineering as an HTL of a planar PSC. The optimal open-circuit voltage has been shown as 1.09 V, the short-circuit current as 20.5 mA/cm^2^, and the fill factor (FF) as 75.7%, and the power conversion efficiency (PCE) as 17.0% of the device. As a result, introducing QDs as a HTL via the ligand engineering can effectively improve the device performance of the PSC. In general, this study has shown that ligand engineering is a candidate for optimizing interfacial characteristic of the PSC.

## Data Availability

Data sharing not applicable.

## Supplementary Materials

The following supporting information can be downloaded at: https://www.mdpi.com/article/10.3390/ma15248902/s1, Figure S1: The tauc’s plot of the QDs with OAM and Octam ligands.
