# Supplementary material for: Planar Perovskite Solar Cells Using Perovskite CsPbI3 Quantum Dots as Efficient Hole Transporting Layers
Source: Materials (Basel). 2022 Dec 13;15(24):8902. doi: 10.3390/ma15248902 (PMC9788023; doi:10.3390/ma15248902)
Supplement: Supplementary file 1 [file materials-15-08902-s001.zip › materials-2023549-supplementary.pdf]

Supporting Information

Planar Perovskite Solar Cells Using Perovskite CsPbI3 Quantum Dots as Efficient Hole Transporting Layers

Tsair-Chun Liang [1], Hsin-Yu Su [1], Sih-An Chen [2,3], Yen-Ju Chen [4], Chung-Yu Chiang [2], Chih-Hsun Chiang [2], Tzung-Ta Kao [1], Lung-Chien Chen [2,*], Chun-Cheng Lin [3,*]

1 Institute of Photonics Engineering, National Kaohsiung University of Science and Technology, Kaohsiung 824005, Taiwan

2 Department of Electro-Optical Engineering, National Taipei University of Technology, Taipei City 106344, Taiwan

3 Department of Mathematic and Physical Sciences, R.O.C. Air Force Academy, Kaohsiung 820008, Taiwan

4 Department of Electronic Engineering, Ming Chi University of Technology, New Taipei City 243303, Taiwan

* Correspondence: ocean@ntut.edu.tw (L.-C.C.); cclincafa@gmail.com (C.-C.L.)

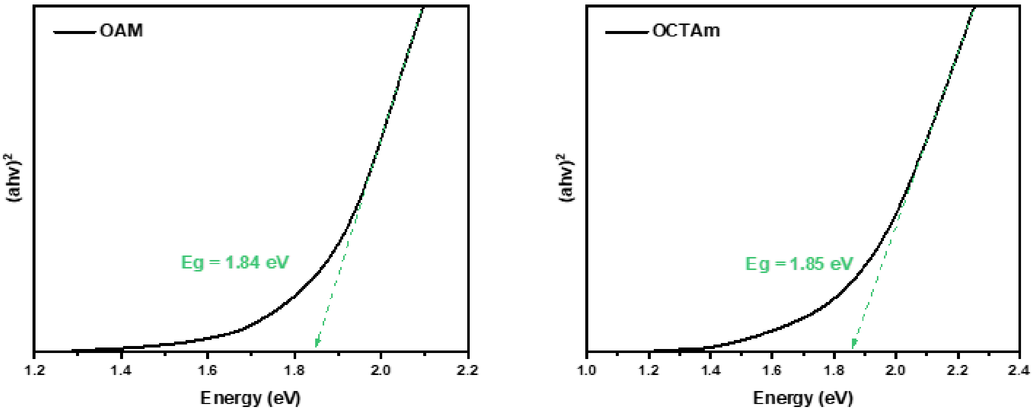

Figure S1. The tauc's plot of the QDs with OAM and Octam ligands.
